# The Role of Maternal Perceptions and Ethnic Background in the Mental Health Help-Seeking Pathway of Adolescent Girls

**DOI:** 10.1007/s10903-012-9621-7

**Published:** 2012-04-22

**Authors:** I. J. E. Flink, T. M. J. Beirens, D. Butte, H. Raat

**Affiliations:** 1 Department of Public Health, Erasmus MC, University Medical Center Rotterdam, Dr Molewaterplein 50, PO box 2040, 3000 CA Rotterdam, The Netherlands; 2 Department of Youth Monitoring, GGD Rotterdam Rijnmond—Municipal Health Service Rotterdam Rijnmond, Schiedamsedijk 95, 3011 EN Rotterdam, The Netherlands

**Keywords:** Ethnic minorities, Mental health, Help-seeking behavior, Parental support, Adolescents

## Abstract

Mothers play a crucial role in the help-seeking pathway of adolescents. This study examined how mothers with different ethnic backgrounds perceive the issue of help-seeking for internalizing problems (e.g. depression) in adolescent girls. Seven focus group discussions were conducted with 41 Dutch, Moroccan and Turkish mothers with a teenage daughter. Discussions were conceptually framed within a model of help-seeking and facilitated by a vignette. The internalizing problems sketched in the vignette were recognized as severe nonetheless; identified long term consequences varied per ethnic group. Negative attitudes towards General Practitioners, inaccessible mental health services and denial by daughters would hamper help-seeking. Fear of negative judgments/gossiping was considered a barrier by Turkish and Moroccan participants. Participants identified themselves and schools as primary sources of help. Turkish participants also named chaplains. To enhance utilization of mental health services by (minority) youth it is important to also address maternal barriers.

## Background

Internalizing problems or problems that are mainly within the self like depression and anxiety [1] greatly affect adolescents. Declining school performance, loss of social relations and substance abuse are some of the frequently reported outcomes of internalizing problems [2, 3]. A vast number of studies have shown that adolescent girls, and particularly those from ethnic minority groups, are more at risk of developing internalizing problems than their male counterparts [4–6]. Early detection and treatment of these problems in adolescent girls is thus of utter importance. A big concern however, is that underutilization of mental health services is high in adolescents [7] and even higher in adolescents from ethnic minority groups in Western countries [8–10].

Research has shown that parents play an important role in the help-seeking pathway of adolescents. In a review by Zwaanswijk et al. [11] parental attitudes, beliefs, educational level and family stress were main determinants of adolescent help-seeking. In adolescent girls, maternal perceptions may be particularly important, as mothers are important figures in their lives. The aim of this study was to examine how mothers with different ethnic backgrounds perceive the issue of help-seeking for internalizing problems experienced by adolescent girls.

## Theoretical Framework

**Fig. 1:**
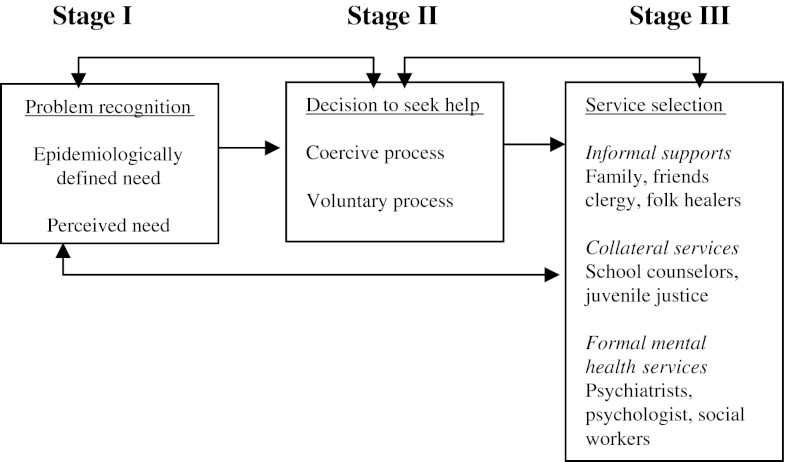
A three-stage model for mental health help-seeking among adolescents developed by Cauce et al. [12]

A model for mental health help-seeking (Fig. 1) developed by Cauce et al. [12], was used for exploring the influence of ethnic background on maternal perceptions of help-seeking. The model consists of three stages of help-seeking that are not necessarily sequential. Stage I in the pathway, referred to as problem recognition, takes into account two types of need: (1) epidemiologically defined need typically assessed according to the Diagnostic and Statistical Manual of Mental Disorders (DSM) criteria, with a clear focus on diagnosis and disease; and (2) perceived need which refers to personal perceptions of need. Stage II, the decision to seek help, consists of a coercive and voluntary process. The coercive process refers to mandated referrals, while the voluntary process refers to voluntary help-seeking which is often influenced by perceptions and attitudes. Stage III, service selection, looks at who the help-seeker turns to after identifying a problem and deciding to seek help. Context and culture are hypothesized to influence all three stages of help seeking [12].

In order to elaborate on the three stages, concepts from the latest version of the Health Belief Model (HBM) by Rosenstock et al. [13] were also considered. The HBM posits that “prevention, screening or, control of ill health conditions are more likely if people regard themselves as susceptible to the condition, if they believe it has serious consequences, if they believe an available action will reduce their susceptibility or severity of the condition and, if the barriers to actions are outweighed by the benefits” [14]. The concepts of perceived severity (how severe are internalizing problems), and perceived barriers and facilitating factors (what hampers or facilitates help-seeking), were taken into account.

## Methods

### Participants

A convenience sample of 41 mothers was recruited via migrant organizations, mosques and schools in a multicultural urban area (Rotterdam, the Netherlands). Recruitment via mental health services was avoided as the focus was on mothers from the general population. Participants were eligible to participate if they were mothers with a teenage daughter aged between 12 and 20 years, and with one of the following ethnic backgrounds: Dutch, Turkish or Moroccan (Turks and Moroccans being the two major ethnic minority groups in the Netherlands). Ethnic background was determined on the basis of the country of birth of the participating mother and her parents [15].

### Data Collection

The Medical Ethical Committee of the Erasmus MC-University Medical Center Rotterdam approved the study. All participants provided their informed consent. The focus group discussions (FGDs) were held in groups of 3–10 participants (clustered according to ethnic background) in schools, centers for women, or mosques. Two FGDs were conducted in Turkish. The remainder of the groups was Dutch spoken however, in these groups a Turkish or Moroccan interpreter was present. Attention was paid to saturation of data. After the seventh FGD had taken place no new themes arose, and data collection ceased. The discussions lasted 1 h and 15 min and the participants received a small incentive for participation. All FGDs were tape-recorded.

### Measures

#### Vignette and Focus Group Guide

A vignette, presenting internalizing problems in an adolescent girl, was employed (Appendix 1). The Focus Group Guide (Appendix 2) was based on the vignette and comprised three sections, corresponding to the three stages of help-seeking. General and probing questions were based on the three stages, perceived severity, barriers and facilitating factors.

#### Questionnaire

The participants completed a questionnaire after the FGDs had ended. This questionnaire included items on background characteristics (e.g. education), and the use of health services in the past year by the participants and their daughters (e.g. mental health services). As psychological well-being may influence maternal perceptions of help-seeking [16], the General Health Questionnaire 12 (GHQ 12) was administered [17]. The GHQ 12 screens for non-psychotic psychiatric disorders in adults [18]. A cut-off score of 15 was used for defining clinical signs of distress as proposed by Goldberg [19].

### Analysis

The recordings from the FGDs were transcribed verbatim and entered into the NVivo software program (version 8) by the primary researcher. Systematic coding of themes and subsequent categories was performed by one coder. Content analysis was conducted by two coders. Major themes were defined beforehand by the two coders and were based on the theoretical framework of the present study. Minor themes were derived from the data [20]. Member checking took place by comparing and discussing the major and minor themes. As a rule, it was decided that themes should be named or discussed by at least two participants per focus group and should arise in all groups, to be considered as an overall finding. If a theme arose exclusively in a Turkish, Moroccan or Dutch group, it would be considered as specific for that ethnic group.

## Results

### Participant Characteristics

Table 1 gives a description of the participants (N = 41). Participants were of Dutch (26.8 %), Moroccan (31.7 %) or Turkish (41.5 %) origin. All non-Dutch participants were first generation immigrants. Age of the participants was 44.3 (standard deviation (SD) 6.1; range 32–64 years) and mean age of their teenage daughters (N = 46) was 15.2 (SD 2.4; range 10–20 years). A majority (77 %) reported to be Muslim. Educational level was based on the highest completed degree of the participant and was considered low when the participant completed no education or primary education, mid when the participant followed vocational or vocational preparatory education and high when the participant followed university preparatory or higher education. Participants mostly had a low level of education (52.5 %), lived with their partner and children (82.9 %) and reported to be housewives (53.7 %). Most had sought help from a general/family practitioner (GP) for themselves and their daughters. The mean score (SD) on the GHQ 12 was 9.0 (5.4). Five participants had a score above 15.0.

**Table 1:** Participant characteristics

	Mean (SD/range)	% (N)
Characteristics of participants (N = 41)		
Age (in years)	44.3 (6.1) (32–64)	
*Ethnicity*		
Dutch		26.8 (11)
Moroccan		31.7 (13)
Turkish		41.5 (17)
*Religious affiliation* ^a^		
Islam		77.1 (27)
Christianity		5.7 (2)
Other		2.9 (1)
No religion		14.3 (5)
*Highest attained educational level* ^b, c^		
Low		52.5 (21)
Mid		35.0 (14)
High		12.5 (5)
*Employment status*		
Full-time		4.9 (2)
Part-time		34.1 (14)
Housewife		53.7 (22)
Unemployed		7.3 (3)
*Current family situation*		
Living with partner and children		82.9 (34)
Living alone with children		17.1 (7)
General Health Questionnaire 12 score^d^	9.1 (5.4) (0–28)	
General Health Questionnaire score >15		15.0 (5)
		N
*Health service use for self (past year)*		
GP		35
Medical specialist		7
Mental health care		4
Social worker		2
*Health service use for daughter (past year)*		
GP		15
Mental health care		3
Medical specialist		4
Social worker		4
Peer help		1
Youth health care		1
Characteristics of teenage daughter (N = 46)		
Age (in years)	15.2 (2.4) (10–20)	

^a^Data missing for 6 participants

^b^Data missing for 1 participant

^c^Educational level low when the participant completed no education or primary education, mid when the participant followed vocational or vocational preparatory education and high when the participant followed university preparatory or higher education

^d^Scores of about 11–12 are typical. Score >15 evidence of distress [14]

### Perceptions of Help-Seeking

#### Stage I: Problem Recognition and Perceived Severity

Most participants recognized that the character in the vignette was dealing with an emotional problem “Well, because she is being bullied and doesn’t feel like going to school, she gets emotional complaints” (Moroccan participant, FGD 1, 2). All participants expressed that the problem was severe “Well, laying in bed all day listening to music, pondering and skipping class is very problematic” (Dutch participant, FGD 2, 3).

##### Ethnic Differences

Ethnic differences were found in the identified consequences of not seeking help. Turkish and Moroccan participants more often indicated that if nothing changed in the character’s situation she could commit suicide “She could go so far that she commits suicide” (Turkish participant, FGD 3, 4). Dutch participants also indicated that suicide was possible however, falling prey of a lover boy (pimp) seemed more realistic to them “I think that there is a bigger chance of her coming into contact with a lover boy than suicide” (Dutch participant, FGD 1, 3).

#### Stage II: Decision to Seek Help

##### Barriers to Help-Seeking

Although the GP was identified as an important gatekeeper to mental health services by all participants “The GP can play a big role because he can tell you where to go and who to choose: a social worker or a psychiatrist” (Moroccan participant, FGD 2), some participants expressed that the GP did not take them seriously “The GPs here are dramatic. They sit behind their computers and ask you to tell them what you have and then tell you that what you have is very common, that everyone has it. That’s how it goes” (Turkish participant, FGD 1, 3).

A majority of the participants expressed that formal mental health services were inaccessible. Dutch participants named long waiting lists and a lack overview of the available mental health services as factors that would hamper them from seeking help from a formal mental health service “My daughter had been to a, uhm, I don’t even know what it’s called. It’s confusing, one day it’s called RIAGG and the other day it’s called something else. But it all takes so long. We started going there 7 or 8 months ago and nothing has actually happened since” (Dutch participant, FGD 1, 5). Some Turkish participants complained about the treatment “I have been to a psychologist. I was asked to go back to my past, my childhood. I had to talk about things that I had done when I was 8, well that doesn’t make me feel any better, psychologically” (Turkish participant, FGD 2, 6).

In all groups, the majority of the participants mentioned that teenage girls often deny that they have a problem. As a result, their mothers find it difficult to identify problems in their daughters and to seek help “I find it a pity that girls of 14, 15 or 16 are usually not open to their moms, no matter how sweet she is. They don’t dare to tell you what’s bothering them. It’s a pity because this way you can’t help them” (Moroccan participant, FGD 2, 7).

##### Ethnic Differences

Most Moroccan and Turkish participants expressed that they feared negative judgments/gossiping when telling anyone outside of the immediate family (e.g. neighbors) about internalizing problems experienced by their daughters “They will immediately start to gossip” “Yes, like have you heard it, she’s done this and that, it’ll go about” (Turkish participant, FGD 3, 4).

##### Facilitating Factors

All participants indicated that a good and trustful bond with your daughter would make it easier for her to come to you when she has a problem “The important thing is having good contact with your daughter and parenting. As in, if there is something you need, come to me. For instance: a problem at school. You need to know about the problem” (Moroccan participant FGD 1, 7).

##### Ethnic Differences

Although all Dutch participants indicated that the school should play a more prominent role in detecting problems and providing feedback concerning problems to the parents, most Dutch participants also expressed that anonymity of a conversation with a professional is of importance for teenage girls, particularly in school settings “If a child clearly states that she does not want them to tell her parents then they shouldn’t” (Dutch participant, FGD 1, 1). Some Turkish and Moroccan participants felt responsible for updating the school if anything was wrong with their daughter “If a child tells the teacher about her problems then there should be some kind of feedback to the mother. I always tell the teacher about problems as well. This way the teacher knows and can keep an eye open” (Turkish participant, FGD 1, 2).

#### Stage III: Selection of Services

##### Informal Services

All participants expressed that an adolescent girl should initially go to her mother so that she can help her further “At the age of 15 she can’t seek help by herself. She needs to go to a psychologist, together with her mother. This way her mother can send her to one” (Turkish participant, FGD 2, 3). After they’ve talked one on one with their daughters, most participants indicated that their next step would be to seek advice from a good friend, a family member or some one from their social network “I would ask advice from a friend or my sister. You need to find a sounding board and ask them whether they think that you’re doing the right thing or whether you are exaggerating for instance” (Dutch participant, FGD 1, 3).

##### School-Based Services

All participants had big expectations concerning the role of teachers and schools in detecting emotional or internalizing problems in children and adolescents “A teacher will notice when a child has problems. She spends more time at school than at home and they’re not blind” (Moroccan participant, FGD 2, 7).

Most participants identified school social workers as the first professional that teenage girls should seek help from because they are the most accessible “My experience is that a child first seeks help at school. She’ll have a conversation there and after that you can go to a GP, this also makes it easier for them to go to a GP” (Dutch participant, FGD 2, 3).

##### Formal Services

If help had to be sought outside school, social workers and GPs were identified as the most accessible professionals by most participants “I hope that, in the end, a social worker is the one that gets her back on track and makes her stronger” (Dutch participant, FGD 1, 3) “I always say; even if you don’t know anything you will always be able to find the GP” (Turkish participant, FGD 1, 3).

Psychiatrists and psychologists were named as the last option by most participants because they thought that it was too big of a step for an adolescent girl “I think that my daughter should come to me first. If we are not able to solve it amongst the two of us then I could look for external help. Possibly a psychiatrist, but only if it’s really necessary. I personally find a psychiatrist a bit too much for a 15 year old” (Moroccan participant, FGD 2, 10).

##### Ethnic Differences

Some Turkish participants also indicated that they would seek help from a chaplain because they are more accessible and easy to talk to than a Dutch professional “I would personally feel more at ease if I talked to my chaplain about these types of problems. She will give me advice” (Turkish participant, FGD 2, 6).

## Discussion

This study examined how mothers with different ethnic backgrounds perceive the issue of help-seeking for internalizing problems experienced by adolescent girls. Findings showed that all participants recognized the severity of internalizing problems. If their daughters were to face internalizing problems, negative attitudes towards GPs (gatekeepers to Dutch mental health care), inaccessible mental health care and denial by daughters, would hamper mothers from seeking professional help. In ethnic minority groups, negative judgments and gossiping were additionally perceived as barriers. Good contact with schools and their daughters would facilitate help-seeking. Participants had a preference for dealing with internalizing problems experienced by their daughters in the informal care setting (e.g. schools).

Before discussing the findings of this study further, it is important to consider its strengths and limitations. A strength is that it includes three different ethnic groups hence facilitating the cross-cultural comparison of perceptions and leading to a better understanding of differences in mental health service utilization among ethnic groups. The first limitation is one of generalizability. The findings of this study are representative for a small sample of mostly low educated Dutch, and first generation Turkish and Moroccan mothers, living in an urban area that were recruited from the general population. It is possible that findings would have been different if higher educated mothers or mothers with more experience with mental health care had been included. Additionally, solely including first generation immigrants did not permit us to look at the influence of acculturation, which has been associated with help-seeking behavior [21, 22]. Regardless, we do believe that parallels can be drawn with non-Western ethnic minority groups in other developed economies as they often face similar situations. Some Turkish and Moroccan participants experienced language barriers in the Dutch spoken groups. Although it was desirable to include these participants, it should be noted that some FGDs may have been dominated by those that mastered the language better. Lastly, this study only focused on ethnic background as this was our determinant of interest. It is however possible that other demographic characteristics like educational level, working status or age, which may be intertwined with the immigrant status and ethnic background, influenced perceptions of maternal help-seeking.

A clear barrier to help-seeking that arose in all FGDs was the denial by daughters when confronted with their emotional state by their mothers. Studies on eating disorders in adolescent girls, also a sensitive topic, showed similar results [23–25]. Another barrier to help-seeking that was perceived in some groups was a negative attitude towards GPs. In the Netherlands, GPs form the gatekeepers to mental health care for adults and youth [26]. If parents are reluctant to go to a GP when dealing with mental health problems (their own or their child’s), they may not seek help at all or will remain in informal care. This is problematic if problems get more serious.

In terms of service selection, participants’ expressed a clear preference for informal care and care at school and were reluctant to seek help from formal mental health care. In general, participants expected their teenage daughters to firstly turn to them when experiencing emotional problems. Hereafter other forms of informal care could be sought. This is in line with previous research [27, 28]. It is noteworthy that participants did not mention consulting their daughter’s friends as a possible source of help even though this is often whom adolescents turn to first [29]. This is in accordance with findings from the Access to Mental Health Care in Children study (AMHC) conducted in Switzerland and Portugal [30, 31]. In our study, the primary reason for choosing informal care over formal care was that participants thought that adolescents are too young to get involved in formal care and therefore, they preferred them to seek help at school. As suggested by other studies [27, 28, 30], it is also possible that the stigmas attached to receiving help from a mental health professional (e.g. a psychiatrist) may be an additional reason for this reluctance.

Ethnic background played a role in maternal perceptions of help-seeking however, most differences were only apparent between the majority and the minority groups. Firstly, Moroccan and Turkish participants perceived different consequences of internalizing problems than Dutch participants. Turkish and Moroccan participants named suicide more often whereas Dutch participants indicated that falling prey to a loverboy (pimp) was more realistic. A plausible explanation for this may lie in parenting style; Moroccan and Turkish girls are raised in a more authoritarian way and often have less freedom of movement and are thus more likely to stay at home than go out on the streets when experiencing internalizing problems [32]. As all Moroccan and Turkish participants in this study reported to be Muslims, it is possible that religious norms influence family values and parenting issues [12].

A barrier that hampered ethnic minority groups to seek help was the fear of negative judgments/gossiping by others. Other researchers have also found this barrier to influence help-seeking for mental health problems in ethnic minority groups [33]. Persisting taboos surrounding mental health problems may be a plausible reason for this fear [33, 34].

There proved to be some discrepancy between the ethnic majority and the ethnic minority groups regarding anonymity and confidentiality in the school setting. Dutch participants clearly indicated that a conversation between a professional and an adolescent should be kept confidential. On the contrary, Turkish and Moroccan participants expressed that it was necessary to regularly inform teachers on how their children were doing and they did not mention confidentiality as important in this regard. Goncalvez et al. [30] also found that, according to teachers, immigrant parents greatly value communication and seek contact with teachers to vent their problems. The perceptions of Turkish and Moroccan mothers regarding confidentiality may, in part, explain why Turkish and Moroccan girls are more reluctant to seek help from, amongst others, their parents [26]. Regardless of these discrepancies, all participants indicated that the school should play a more prominent role in detecting and treating mental health problems in pupils and contacting parents when this proved necessary.

## Conclusions

This study identified maternal perceptions of mental health help-seeking for adolescent girls, taking ethnic background into account. To enhance utilization of mental health services, it will be beneficial to increase parental knowledge of internalizing problems so that problems can be detected and treated in an early stage despite denial by adolescent girls. Attention should also be paid to changing maternal attitudes towards GPs or other gatekeepers to mental health care. In ethnic minority groups, mental health problems should be made more discussable so that taboos can be tackled. Lastly, the school could play a more prominent role in detecting and treating internalizing problems nevertheless, confidentiality should be considered.

## Appendix 1: Vignette

### Dina, aged 15 years

Dina is from a family with four children. For some time now things haven’t been going well for her. She cries a lot and doesn’t sleep well. For nights in a row she lies worrying: about school, about boys and about the situation at home. At school, her classmates bully her and laugh at her. She thinks it’s because she always blushes during presentations. Also, her grades are not good: there’s even a possibility she won’t pass this year. Her parents don’t agree with this and have spoken to her teacher several times. At home they’re always moaning about her low grades. Her brothers and sisters don’t interfere with her life. Dina thinks they’re better at everything anyway.

For some time, she hasn’t been in the mood for school. She has skipped some classes already, preferring to stay in bed all day, to listen to music and to ponder.

## Appendix 2: Questions Used as Prompts During the Focus Group Discussions

1. What do you think is wrong with Dina?
2. Do you think it’s serious/severe?
3. What could happen to Dina if she doesn’t seek help?
4. Why do you think she has these problems?
5. (for Moroccan and Turkish groups only) Do you think that a Moroccan or Turkish girl could also have these kinds of problems?
6. How would your friends react if your daughter had such a problem?
7. How would other people from your environment react (i.e. teachers, other family members, and friends)?
8. How important do you find it is for Dina to seek help?
9. Imagine your daughter is dealing with the same problems, who would you approach for help?
10. Which obstacles would hold you back from seeking help for her?
11. What would make seeking help easier?
12. Do you think Dina can seek help by herself?
13. Do you know anyone that has helped her daughter to seek help for problems like these?
14. Can you tell us a little more about it?
15. Should Dina have sought help earlier?
16. When should she have done that?
17. Can Dina solve her problems by herself?
18. How can friends help?
19. How can parents help?
20. How can teachers help?
21. How can professionals (GPs, social workers, psychologists) help?
22. (for Moroccan and Turkish groups only) Say Dina is a Moroccan or Turkish girl, are there any religious gatekeepers or chaplains she can turn to?
23. Are some groups more vulnerable for these kinds of problems?
24. What do you think causes these problems?
